# Cadmium Removal from Contaminated Water Using Polyelectrolyte-Coated Industrial Waste Fly Ash

**DOI:** 10.1155/2017/7298351

**Published:** 2017-06-07

**Authors:** Fatai A. Olabemiwo, Bassam S. Tawabini, Faheemuddin Patel, Tajudeen A. Oyehan, Mazen Khaled, Tahar Laoui

**Affiliations:** ^1^Geosciences Department, College of Petroleum & Geosciences, King Fahd University of Petroleum & Minerals (KFUPM), Dhahran 31261, Saudi Arabia; ^2^Mechanical Engineering Department, KFUPM, Dhahran 31261, Saudi Arabia; ^3^Chemistry Department, KFUPM, Dhahran 31261, Saudi Arabia

## Abstract

Fly ash (FA) is a major industrial waste generated from power stations that add extra cost for proper disposal. Recent research efforts have consequently focused on developing ways to make use of FA in environmentally sound applications. This study, therefore, investigates the potential ability of raw fly ash (RFA) and polyelectrolyte-coated fly ash (PEFA) to remove cadmium (Cd) from polluted water. Using layer-by-layer approach, functionalized fly ash was coated with 20 layers from 0.03% (v/v) of cationic poly(diallyldimethylammonium chloride) (PDADMAC) and anionic polystyrene sulfonate (PSS) solutions. Both surface morphology and chemical composition of the adsorbent (PEFA) were characterized using Field-Emission Scanning Electron Microscope (FE-SEM), X-Ray Diffraction (XRD), Fourier-Transform Infrared (FTIR), and X-Ray Fluorescence (XRF) techniques. The effects of pH, adsorbent dosage, contact time, initial contaminant concentration, and mixing rate of the adsorption of Cd were also studied in batch mode experiments. Results of the study revealed that a 4.0 g/L dosage of PEFA removed around 99% of 2.0 mg/L of Cd in 15 min at 150 rpm compared to only 27% Cd removal achieved by RFA under the same conditions. Results also showed that adsorption by PEFA followed both Langmuir and Freundlich models with correlation coefficients of 98% and 99%, respectively.

## 1. Introduction

In today's world, one issue of major concern is water pollution as the quality of water available for consumption greatly affects the health and wellbeing of humans and animals. Factors like industrialization, agricultural activities, urbanization, and population increase among others are likely reasons for water quality depreciation [1, 2]. The constant discharge of different pollutants such as organic compounds and heavy metals into the environment is causing growing concern to the entire world. Unlike most organic contaminants, heavy metals are mainly problematic because they accumulate in the tissues of living organisms and do not biodegrade, thereby leading to countless threats to the ecological environments and wellbeing of humans at large [3]. Majorly known heavy metals primarily consist of cadmium, chromium, mercury, lead, cobalt, nickel, and so forth; these metallic ions are toxic and pose severe effects on human health.

Cadmium (Cd) which is a deadly heavy metal of work-related and environmental worry has been recognized as a substance that is teratogenic and carcinogenic to human. The allowable limit for Cd in drinking water is set at 3.0 ppb by World Health Organization (WHO) [4]. If ingested beyond the limit, it would affect the kidney or probably damage it. Common ways via which Cd get leached to the environment include industrial processes like smelting, alloy manufacturing, and pesticide and anthropogenic activities such as improper disposal of cigarette, unused paints, fertilizers, and Ni/Cd batteries [5]. Therefore, the removal of this heavy metal from contaminated water has become a task of paramount importance. For that, numerous methods such as membrane separation, ion exchange, coagulation, softening, solvent extraction, and adsorption have been employed [6]. Some of these techniques are effective but are not widely applicable to different pollutants and also generate chemical waste. Application of these techniques relies on cadmium concentration and associated costs [7]. Mahvi & Bazrafshan (2007) applied electrocoagulation to remediate cadmium using Al electrode. Simulated wastewater of different concentrations of cadmium was filled in a tank and their removal was measured at different pH (3, 7, and 10) and at electric potential range of 20, 30, and 40 volts. Their investigation showed that initial pH was lower than the final pH value [8]. Numerous studies have used the adsorption mechanism for heavy metals removal using activated carbon owing to its very good adsorption features but with comparatively high operating cost [9, 10]. Therefore, the need to develop low-cost adsorbents for heavy metals removal from aqueous solution has greatly increased. Adsorbents such as* Setaria* grass [11], sawdust [12], zeolite [13], clay [9], biomass [14, 15], and fly ash [6, 16–19] have been used.

Fly ash (FA) is one of the major wastes from power stations that cannot be cheaply disposed of. Recent research efforts have consequently focused on developing ways to make use of FA in applications that are friendly to the environment. Apart from its limited applications in cement and concrete industries, fly ash alternative use/reuse in environmental study takes advantage of its reasonable adsorptive property for some water pollutants. Al-Khaldi et al. conducted a comparative study on Cd adsorption using activated carbon, CNT, CNF, and fly ash. They found out that, at pH 7 in 120 min with 50 mg and 150 rpm, percent removal of 95, 27, 34, and 38% was attained for fly ash, CNT, CNF, and activated carbon, respectively [7]. However, the efficiency of the FA for removing water pollutants was limited in a few previous studies and, therefore, there is a need to improve its adsorption efficiency [18]. One way to achieve this objective could be to coat the surface with polyelectrolytes which enhanced the adsorptive capacity of several adsorbent materials [18]. Literature search showed that no work has been conducted to assess the efficiency of FA to remove Cd from water after being coated with layers of polyelectrolytes which is the main aim of this work. As fly ash is cheaper compared to other adsorbent materials, any improvement in the efficiency of polyelectrolyte-coated fly ash in the removal of heavy metal ions from water gives it an advantage over other adsorbent materials.

Polyelectrolytes are charged organic polymers, which are soluble in water and are formed from monomers of different kinds. These polymers could be cationic or anionic depending on the functional ionic group. They are used in many applications such as water purification and paper production [20]. Examples of polyelectrolytes include poly(diallyldimethylammonium chloride) (PDADMAC), polystyrene sulfonate (PSS), and polyethylenimine (PEI). Studies had been made to modify adsorbents with polyelectrolytes. Zhang et al. [21] modified multiwalled CNT with PDADMAC for chromium adsorption of which 32% removal was achieved at pH 6. Huang et al. [22] successfully applied silica-coated Fe_3_O_4_ functionalized with c-mercaptopropyltrimethoxysilane for extraction of Cu^2+^, Hg^2+^, Cd^2+^, and Pb^2+^ in a varied pH range and even in the presence of foreign ions acting as interferents such as Al^3+^, Fe^3+^, and Cl^−^. Stanton et al. [23] showed that alternating polyelectrolyte deposition on porous supports can yield nanofiltration membranes allowing high water flux along with selective ion transport by using pairs of poly(styrene sulfonate)/poly(allylamine hydrochloride) on porous alumina.

**Figure 1:**
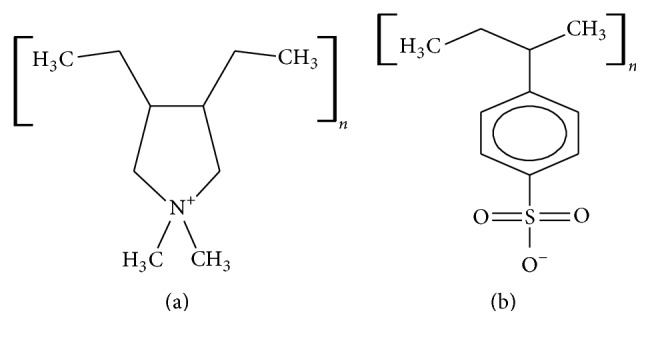
Chemical structures of (a) poly(diallyldimethylammonium chloride (PDADMAC) and (b) polystyrene sulfonate (PSS).

The aim of this study is to explore the potential efficiency of fly ash to remove Cd ions from contaminated water and to evaluate the effect of acid treatment and polyelectrolyte coating of fly ash on removal efficiency. Moreover, the effects of experimental parameters such as adsorbent dose, contact time, pH, mixing rate, initial concentration, and temperature on the Cd ion removal efficiency were also deduced. Thermodynamic parameters like enthalpy, entropy, and Gibbs free energy were also investigated. The acid treatment of fly ash was done using HNO_3_ and the polyelectrolyte coating of fly ash is done by layer-by-layer (LBL) deposition of two electrolytes, namely, PDADMAC and PSS, solutions on acid-treated fly ash (AFA). The Cd ion removal efficiency of adsorbents was measured using batch adsorption experiments. The sorption kinetics of Cd on the adsorbents were investigated using Langmuir and Freundlich isotherm models.  Figure 1 shows the chemical structures of PSS and PDADMAC.

## 2. Materials and Methods

### 2.1. Chemicals/Stock Solution

All chemicals and solvents used were of analytical grade. PDADMAC (Mw: 200,000–350,000 kg/mol.) and PSS (Mw: 70,000 kg/mol.) were commercially acquired and used. Deionized (DI) water was generated in real time from Milli-Q Ultrapure water system (Millipore). Working standard solutions were prepared from stock Cadmium ICP Standard Solution supplied by ULTRA Scientific (USA) by serial progressive dilutions with deionized water. The prepared solutions were stirred for 30 mins with a magnetic stirrer to ensure homogeneity. The pH of the solutions was adjusted using either 0.1 M HNO_3_ or 0.1 M NaOH solution. Buffer solutions were added as required in order to keep constant pH during the experiment.

### 2.2. Adsorbent Preparation

The fly ash used in this study was obtained from a local power plant in the Eastern Province of Saudi Arabia. In this plant, raw fly ash is generated from the combustion of heavy liquid fuel and collected by electrostatic precipitation technique. This raw fly ash (RFA) was processed further to produce acid-treated fly ash (AFA) and polyelectrolyte-coated fly ash (PEFA).

#### 2.2.1. Acid Treatment of Fly Ash

150 g of fly ash materials was soaked in 1000 mL DI water and stirred for 2 h. After stirring, the mixture was allowed to settle for 10 min before the water was decanted and the procedure was repeated 3 times which gives a slurry phase, which was subsequently dried in the oven at 80°C temperature for 12 h and stored until used for the batch treatment experiments. 100 g of washed fly ash was soaked in 300 mL of 1 M HNO_3_. The mixture was refluxed at 110°C temperature for 24 h. The acid was allowed to evaporate at 60°C, after which the reaction mixture was diluted with 500 mL DI water until the pH of the filtrate becomes neutral. The residue was then dried in the oven at 105°C for 72 h [24–29].

#### 2.2.2. Layer-by-Layer Deposition (Polyelectrolyte Coating of Fly Ash)

**Figure 2:**
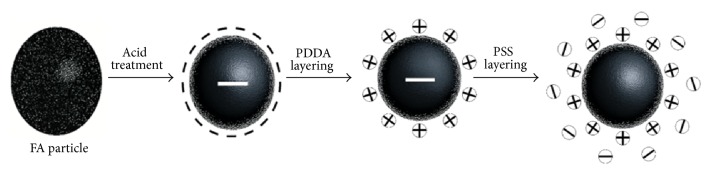
Schematic illustration of acid treatment and polyelectrolytes coating of fly ash (FA).

Polyelectrolyte coating of fly ash (PEFA) was prepared by coating the acid-treated fly ash (AFA) with polyelectrolyte (PE) using modified procedure of layer-by-layer method described by Li et al. [30]. Succinctly, the solutions used were prepared by dissolving 3 mL of poly(diallyldimethylammonium chloride) (PDADMAC) or polystyrene sulfonate (PSS) in 1000 mL of water; the solutions were stirred with a stirrer to ensure a homogenous mixture. The layering then followed Li et al.'s [30] procedure but without the addition of NaCl to ensure the formation of thinly coated PE layers as illustrated in Figure 2. The procedure was repeated until the desired number of layers was attained, that is (PDADMAC/PSS-FA)_*n*_, where *n* could be 1,2, 3,4, 5,…, 20.

### 2.3. Characterization of FA Adsorbents

Characterization of adsorbents (RFA, AFA, and PEFA) surface morphology was conducted to understand elemental, mineralogical, and functional group composition. Scanning electron microscopy (SEM) micrographs were documented using FESEM (JSM-5900LV) fitted with an energy disperse X-ray spectroscopy (EDX) detector model X-max. Functional groups were determined using a Perkin-Elmer 16 FPC FTIR spectrometer with the aid of KBr pellets and spectra were generated in the region of 600–4000 cm^−1^ wavenumber. Thermogravimetric analysis (TGA) was carried out using thermal analyzer (STA 449 F3 Jupiter) by Netzsch, Germany. The analysis was conducted in air at a distinct temperature ramped at 10°C per min to 900°C [6]. Phase analysis of the adsorbents was evaluated using D8 ADVANCE X-ray Diffractometer manufactured by BRUKER (Germany).

### 2.4. Batch Adsorption Studies

Batch mode adsorption studies were conducted at room temperature in 100 mL Erlenmeyer flasks covered with aluminium foil to avoid contamination. Effects of pH, contact time, adsorbent dosage, mixing rate, initial concentration, and temperature were investigated. Analysis of initial and final concentration of Cd ions was conducted using Optima 8000® ICP-OES Spectrometer (Perkin-Elmer, USA). The percent removal, as well as adsorption capacity of metal ions, was calculated with the following equations:

(1) %  removal=Ci−CeCi×100adsorption  capacity,  qemg/g=Ci−CeMs×V,

where *C*_*i*_ is the metal ion initial concentration in solution (mg/L), *C*_*e*_ is the final concentration of adsorbate ion in solution (mg/L), *V* is the total volume of solution (L), and *M*_*s*_ is adsorbent dosage.

Mean values of 5 replicates were used for data analysis to ensure reproducibility; relative standard deviation (RSD) was in the range of ±3–5%. The precision of the standard solution for analysis was better than 3%.

### 2.5. Adsorption Isotherm Model

The descriptions of adsorption behaviors are usually provided by mathematical models known as the adsorption isotherm models [6]. The distribution of adsorbate molecules between the liquid phase and a solid phase at equilibrium state can be indicated by the adsorption isotherm [25]. In this study, Langmuir and Freundlich isotherm models were employed to assess the adsorption behavior of polyelectrolyte-coated fly ash (PEFA) for Cd ion removal in an aqueous medium. Langmuir isotherm model explains the monolayer adsorption, suggesting that adsorbent materials have finite capacity, considered as the equilibrium state beyond which no further adsorption takes place [31]. The existence of specific homogeneous sites within the adsorbent at which adsorption occurs is the main assumption of this model. The Freundlich isotherm model also explains the adsorptive behavior of the adsorbent material. Adsorption on a heterogeneous surface with the interaction between adsorbate molecules is the main application of this model. The Langmuir and Freundlich isotherms are expressed by the following equation:

(2) $$\begin{array}{c} Q_{e}=\frac{Q_{max}K_{L}C_{e}}{1+K_{L}C_{e}}. \end{array}$$

The above equation can be linearized to

(3) $$\begin{array}{c} \frac{1}{Q_{e}}=\frac{1}{Q_{max}K_{L}C_{e}}+\frac{1}{Q_{max}}. \end{array}$$

From (2), *C*_*e*_ is the equilibrium of Cd concentration (mg/L); *Q*_*e*_ is the amount of Cd (mg) adsorbed per gram of the adsorbent at equilibrium (mg/g); *Q*_max_ is the theoretical maximum adsorption capacity (mg/g); and *K*_*L*_ is the Langmuir isotherm constant (L/mg). A linear plot of 1/*Q*_*e*_ versus 1/*C*_*e*_ can be used to obtain the values of *Q*_max_ and *K*_*L*_ from slope and intercept, respectively.

(4) $$\begin{array}{c} Q_{e}=K_{f}{C_{e}}^{1/n}. \end{array}$$

The above equation can be linearized to

(5) $$\begin{array}{c} {\ln Q}_{e}=\ln K_{f}+\frac{1}{n}\ln C_{e}. \end{array}$$

From the equation above, *K*_*f*_ is the Freundlich adsorption constant related to the adsorption capacity [(mg/g) (L/mg)], while the remaining parameters (*Q*_*e*_ and *C*_*e*_) were described above. A linear plot of ln⁡*Q*_*e*_ versus ln⁡*C*_*e*_ can be used to obtain the values of *K*_*f*_ and *n* from intercept and slope, respectively.

### 2.6. Kinetic Modelling Studies

The adsorption of Cd (II) was analyzed using different kinetic models like pseudo-first-order model, pseudo-second-order model, and Weber intraparticle diffusion expressed in the following equations:

(6) log⁡qe−qtqe=−KLt2.303

(7) 1qe−qt=1qe+Kt

(8) tqt=12Ksqe2+tqe

(9) qt=Kidt1/2+C.

In the equations above, *q*_*e*_ and *q*_*t*_ are amounts of Cd adsorbed (mg/g) at equilibrium and at a given time, *t* (min), respectively. *K*_*L*_ is the pseudo-first-order rate constant sorption (min^−1^). *K*_*s*_ and *K* are pseudo-second-order and second-order adsorption rate constants (g·mg^−1^·min^−1^). *K*_id_, *t*^1/2^, and *C* are intraparticle diffusion rate constant (mg/g·min^−1^), square root of time (min)^1/2^, and intercept, respectively. The constants (*K*_*L*_, *K*_*s*_, and *K*) can be determined from the slopes of linear plots of log⁡(*q*_*e*_ − *q*_*t*_) against *t*, *t*/*q*_*t*_ against *t*, and 1/(*q*_*e*_ − *q*_*t*_) against *t*, where *q*_*e*_ can be determined from the intercept data of pseudo-second-order and second-order rate equations.

## 3. Results and Discussions

### 3.1. Material Characterization

#### 3.1.1. Surface Morphology

**Figure 3:**
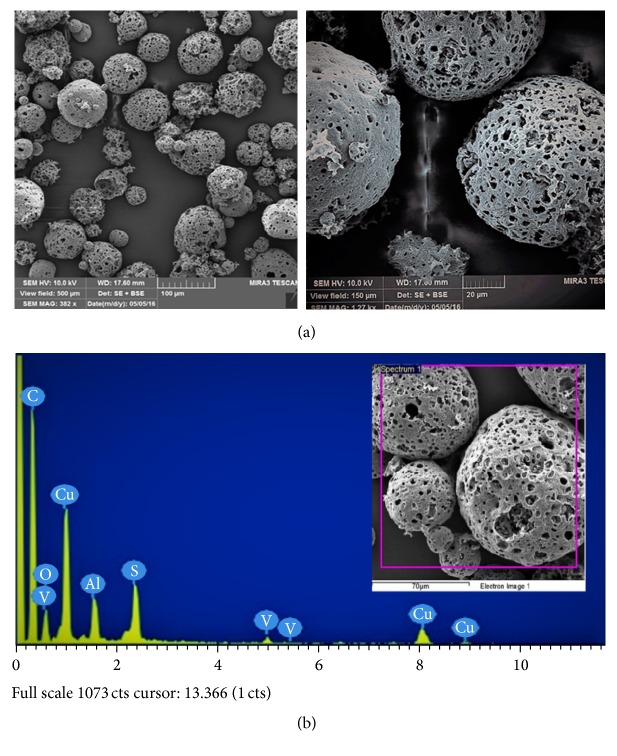
Raw FA (a) SEM micrographs (view field: 500 and 150 *µ*m; voltage: 10 kV; resolution: 382x and 1.27 kx); (b) EDX spectrum.

**Figure 4:**
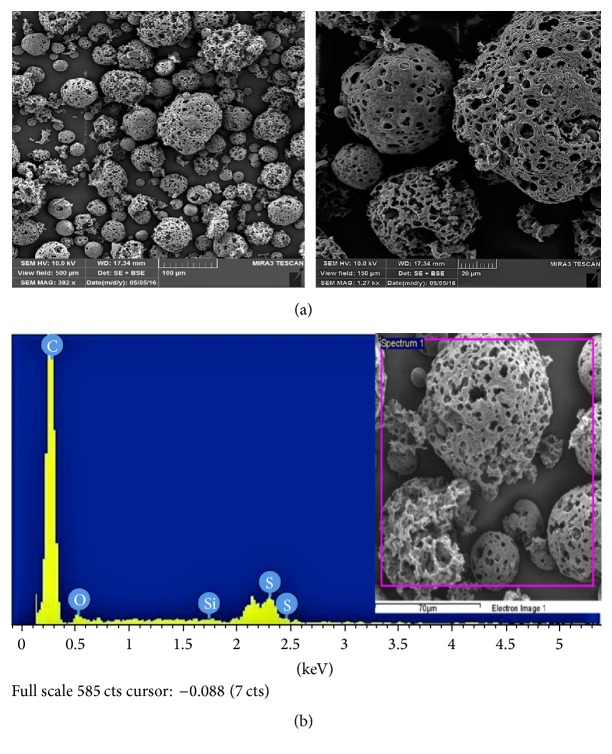
Acid-treated FA (a) SEM micrographs (view field: 500 and 150 *µ*m; voltage: 10 kV; resolution: 382x and 1.27 kx); (b) EDX spectrum.

**Figure 5:**
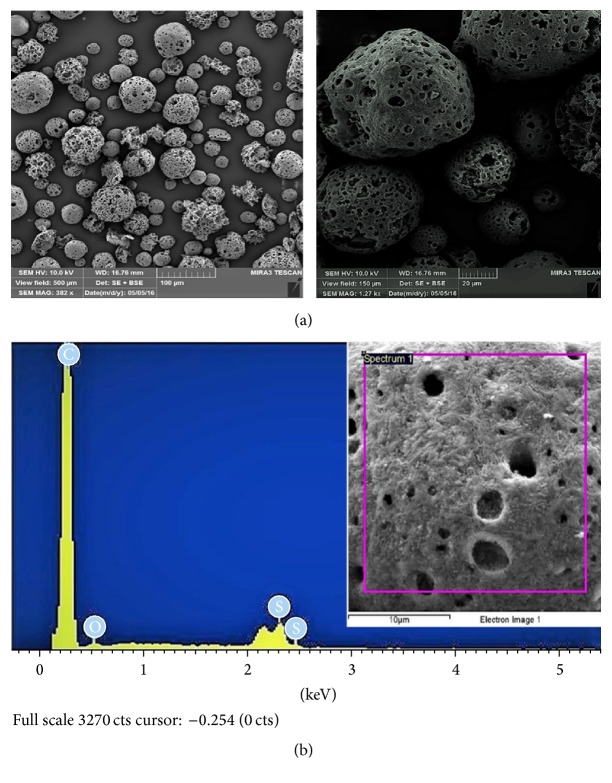
Polyelectrolyte-coated FA (a) SEM micrographs (view field: 500 and 150 *µ*m; voltage: 10 kV; resolution: 382x and 1.27 kx); (b) EDX spectrum.

Surface morphology of raw fly ash (RFA), acid-treated FA (AFA), and polyelectrolyte-coated FA (PEFA) was examined with the aid of Field-Emission Scanning Electron Microscopy (FESEM) and energy disperse X-ray spectrometry (EDX). The surface morphologies of the RFA, AFA, and PEFA are presented in Figures 3, 4, and 5, respectively.

Figure 3(a) shows that the RFA has sizes that range from 50 to 500 microns. Elemental composition revealed by EDX spectra in Figure 3(b) shows that carbon (C) has 72%, oxygen has 16.6%, and the remaining elements, silicon (Si), copper (Cu), vanadium (V), aluminium (Al), and sulphur (S), were found to have 0.2, 6.0, 0.7, 2.0, and 2.8% composition, respectively.

When RFA was treated with nitric acid (HNO_3_), most of the heavy metals impurities present in the as-received raw fly ash were removed as shown in the EDX spectrum in Figure 4(b). Moreover, it was observed that more pores were visible as a result of the treatment with nitric acid as shown in Figure 4(a) compared to raw fly ash shown in Figure 3(a). The spectrum in Figure 4(b) reveals that the carbon content increased from 72 to 92% and also reveals an increase in the silicon content from 0.2 to 0.4.

Figure 5(a) shows the morphology of polyelectrolyte-coated fly ash (PEFA) along with its elemental composition in Figure 5(b). The SEM image shows that a thin pore linen was coated with PDADMAC-PSS and the EDX spectrum shows an increase in the sulphur content (2.77–5.03%) of the fly ash upon coating with polyelectrolyte which might be due to the component of the polymer that has a polystyrene sulfonate compound (i.e., PSS) in its composition.

#### 3.1.2. Elemental Analysis by X-Ray Fluorescence (XRF) Analysis

XRF analysis was carried out to determine the actual elemental composition of the RFA, AFA, and PEFA adsorbents. The results, as summarised in Table 1, identified the presence of some trace metals like vanadium, manganese, iron, nickel, zinc, and molybdenum with their percentage compositions. It was observed that RFA has no silicon content but has high sulphur content of 51% composition which could be attributed to the fact that the fly ash used in this study is an oil fly ash, received from local power plants operating on liquid fuel. This type of fly ash is usually characterized by low silicon and aluminium contents [32, 33]. As could be inferred from the EDX spectrum, the fly ash has a high carbonaceous content which is not commonly found at that rate in coal fly ash with high silicon and aluminium contents [32, 34]. After treatment with acid (AFA), the fly ash trace metal content was reduced to a nonsignificant value, whereas sulphur content increased from 51% to 86% as shown in Table 1. In PEFA, trace metals were either absent or not present in detectable quantity but sulphur content increased to 92.5% which might be a result of additional sulphate group present in the polymer used for coating.

#### 3.1.3. FTIR (Fourier-Transform Infrared) Spectroscopy Analysis

**Figure 6:**
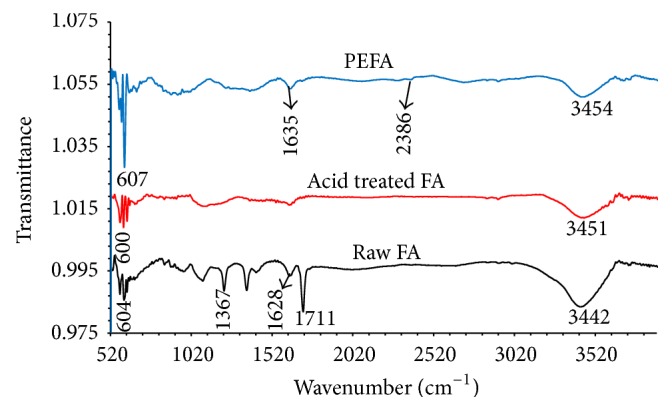
IR spectra of RFA, AFA, and PEFA.

FTIR technique was used to ascertain the functional groups present in RFA, AFA, and PEFA surface. The samples were scanned from 500 to 4000 cm^−1^ and the intensity of peaks in the IR spectra was observed.  Figure 6 shows FTIR spectra for RFA, AFA, and PEFA. The raw fly ash shows a mildly prominent peak at 604 cm^−1^ as a result of the naturally occurring C-S bond [35]. There was a prominent peak at 1367 cm^−1^ as a result of skeletal vibration of a C-C bond [26]. A peak was observed at 1628 cm^−1^, which indicates the presence of C=C functional group of an alkene [28, 36]. A sharp peak at 1711 cm^−1^ represents C=O in ester group as noted by Shawabkeh et al. [27]. A broad trough was observed at 3436 cm^−1^ as a result of O-H stretching of alcoholic groups [27, 37]. However, after treatment with an acid (HNO_3_), peaks were only seen at lower and higher region of the spectra; this might be a result of the bond breaking due to reactions between the acid and fly ash particles. After coating the AFA with polyelectrolytes (PDADMAC and PSS), a more prominent and sharp peak was observed at 607 cm^−1^ of PEFA which is evident of the presence of more C-S functional groups. The peak of C=C reappeared at 1635 cm^−1^ [29, 38]. Additionally, there was C-N peak at 2386 cm^−1^, which indicates the presence of the polyelectrolytes PDADAMAC on the fly ash [39, 40]. The peaks observed at 3442 and 3451 cm^−1^ of the RFA and AFA spectra, respectively, were also observed at 3454 cm^−1^ in PEFA indicating the presence of carboxylic acid O-H functional groups.

#### 3.1.4. Phase Identification by X-Ray Diffraction (XRD)

**Figure 7:**
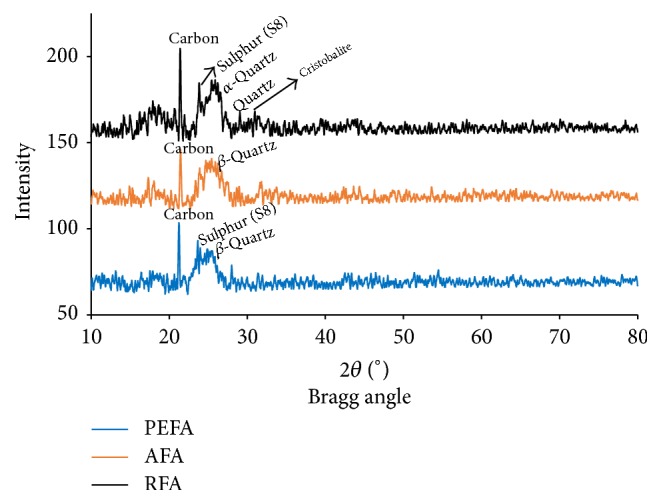
XRD spectra of RFA, AFA, and PEFA.

The mineralogical compositions of RFA, AFA, and PEFA were studied using X-ray diffractometer. Overall, the XRD spectra shown in Figure 7 show the presence of carbon, sulphur, *α*-quartz (low quartz content), *β*-quartz (high quartz content), and cristobalite. A prominent peak was observed at 21.6° 2*θ*; this confirms the presence of highly concentrated carbon. The amorphous phase between 22° and 28° 2*θ* contains sulphur and quartz, respectively. A small peak of cristobalite at 31.4° 2*θ* was observed. Carbon was very prominent through the prepared adsorbents (RFA, AFA, and PEFA). One significant observation was the presence of *β*-quartz at 27.4° 2*θ* in both AFA and PEFA, respectively. No significant peaks were observed after 40° 2*θ*, indicating the presence of amorphous carbon. The XRD pattern of this material can be attributed to that of carbon black or oil fly ash which are both amorphous. Also, the crystalline structure of oil fly ash is known to consist of carbon and metallic sulphur in the amorphous state [41]. Hence, the fly ash used to prepare PEFA in this study can be referred to as oil fly ash as confirmed from the XRD spectrum.

#### 3.1.5. Thermogravimetric Analysis (TGA)

**Figure 8:**
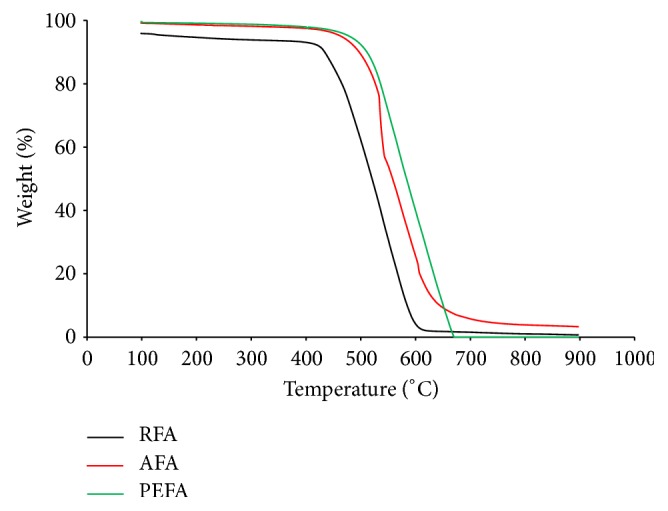
Thermogravimetric (TG) measurements of RFA, AFA, and PEFA.

Thermogravimetric analysis was performed to measure the thermal stability and purity of adsorbents.  Figure 8 displays the thermograms of RFA, AFA, and PEFA. All samples analyzed exhibit similar curves and do not contain adsorbed water. Due to volatilization/decomposition of organic or inorganic substances, a 2% weight loss was observed between 100 and 470°C in RFA. Dramatic weight losses of 84% for RFA between 470 and 600°C, 80% for AFA at temperature range of 580–630°C, and 97% for PEFA between 580 and 670°C can be attributed to the phenomenon of gas generation (CO_2_ and CO) upon pyrolysis [42]. Among the three adsorbents, AFA seems to be more thermally stable than the rest with a residual of approximately 5%. Other samples burn off almost completely before the maximum set temperature of 900°C.

### 3.2. Removal of Cadmium

#### 3.2.1. Effect of pH

**Figure 9:**
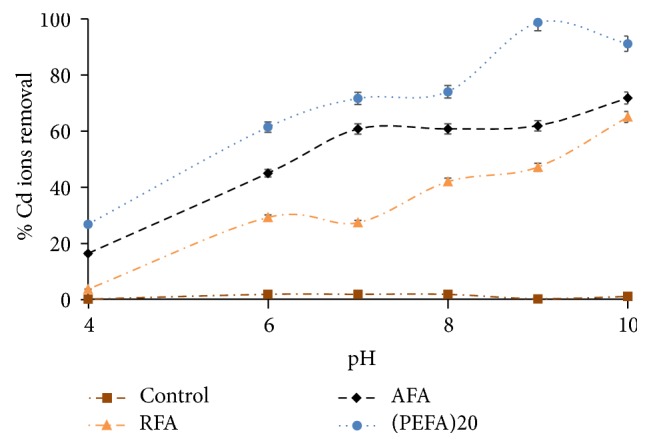
The influence of pH on the removal efficiency of Cd (II) ions on FA based adsorbents (RFA, AFA, and PEFA) as a function of 2 mg/L metal ion concentration, 4 g/L adsorbent dosage, 50 mL volume of aqueous solution, mixing rate of 150 rpm, 15 min contact time, and 298 K temperature.

Generally, metal adsorption consists of a multifaceted mechanism of ion exchange, metal chelating with numerous anionic functional groups, physical forces sorption, and trapping of ions in the interior sphere of adsorbents architectural network [9]. Different forms of Cd species occur in deionized water as Cd^2+^, Cd(OH)_2(s)_, and Cd(OH)^+^ [43]. pH was a leading factor affecting Cd (II) ion removal under the investigated conditions. Nonetheless, Cd^2+^ often exists as a complex [Cd(H_2_O)_6_]^2+^ at low pH and also as prevailing species [44]. With a specific focus on PEFA, the adsorption of Cd (II) ions by RFA, AFA, and PEFA was investigated at pH 4–10 to fix the optimum pH removal.  Figure 9 illustrates an increase in Cd (II) removal efficiency with increased pH in aqueous solution with other parameters fixed at 2 mg/L of metal ion concentration, 4 g/L of adsorbent dosage, 50 mL volume of aqueous solution, mixing rate of 150 rpm, contact time of 15 min, and temperature of 298 K. Maximum sorption of Cd ion was attained at pH 9 due to the fact that in acidic medium Cd (II) ion sorption is low as a result of available large number of hydrogen ions (H^+^) which outcompete Cd ions for active sites. However, as the pH increases, the number of positively charged ions available for active sites reduces with a rise in negatively charged ion for binding [38]. Moreover, the sudden increase and decrease in the removal efficiency as observed in Figure 9 suggest an elaborate process of exchanging ions, sorption driven by physical forces, metal chelation, and trapping of ions in the internal sphere of the structural arrangement of the adsorbents [45].

#### 3.2.2. Effect of Contact Time

**Figure 10:**
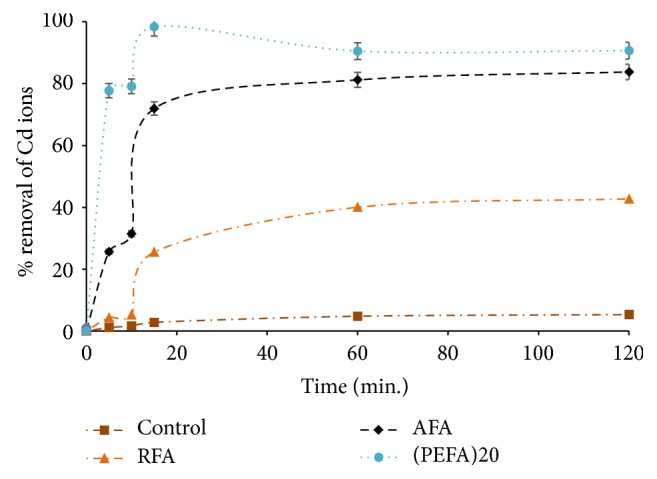
The influence of contact time on the removal efficiency of Cd (II) ions on FA based adsorbents (RFA, AFA, and PEFA) as a function of pH value of 9, 2 mg/L metal ion concentration, 4 g/L adsorbent dosage, mixing rate of 150 rpm, 50 mL volume of aqueous solution, and 298 K temperature.

Contact time is the time required for equilibrium to be attained in the process of adsorption when no substantial variations are detected in adsorptive concentration after a definite period of time [38]. It hinges on the surface features of the adsorbent in question. To find the optimum contact time for Cd (II) ions uptake, varying contact times from 5 min to 2 h were studied from aqueous solutions of 2 mg/L Cd (II) ions concentration, adsorbent mass of 4 g/L, pH value of 9, mixing rate of 150 rpm, and 298 K temperature. The results obtained indicated that at first there was rapid adsorption of Cd (II) ions for PEFA with 98% removal and a gradual decrease to attain equilibrium in 2 h as shown in Figure 10. Initial fast adsorption for this adsorbent might be a result of rich active sites on the adsorbent surface which become filled up with increasing time and turn out to be saturated [9, 38, 46]. Moreover, the decline in the removal efficiency could be attributed to the presence of metal impurities (V, Mn, Fe, Ni, Mo, and Zn) as revealed by XRF in Table 1, which might have occupied the active site needed for sorption. For this study, optimum contact time was chosen to be 15 min as maximum Cd ions removal was reached at this time. Percent removal for RFA and AFA was 25 and 72%, respectively, at the chosen optimum contact time.

#### 3.2.3. Effect of Adsorbent Dosage

**Figure 11:**
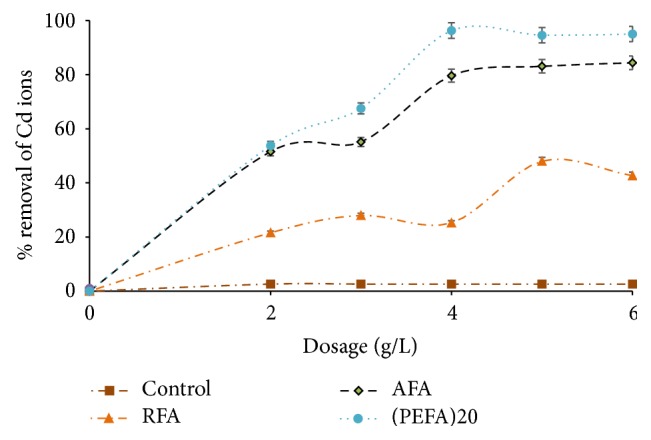
The influence of Adsorbent dose on the removal efficiency of Cd (II) ions on FA based adsorbents (RFA, AFA, and PEFA) as a function of pH value of 9, 2 mg/L metal ion concentration, 15 min contact time, mixing rate of 150 rpm, 50 mL volume of aqueous solution, and 298 K temperature.

The mass of adsorbent has an effect on the active site available for binding of Cd (II) ions in aqueous solution [25, 46]. In this study, batch mode experiments were conducted by applying varying quantities of RFA, AFA, and PEFA from 1 to 6 g/L at pH value of 9, 2 mg/L metal ion concentration, 150 rpm mixing rate, 15 min contact time, 50 mL volume aqueous solution, and 298 K temperature. As illustrated in Figure 11, Cd (II) ion sorption rises with an increase in dose of adsorbents up till 4 g/L and there was little or no significant adsorption for remaining dosage. Sorption increase with an increase in dose of adsorbent could be attributed to surface area increase, the rise in the exchange site ability of the ion, and an increase in active sites [18, 19, 38, 47]. PEFA reaches optimum at 4 g/L unlike RFA and AFA with 5 g/L and 6 g/L as well as removal efficiency of 48 and 84%, respectively. Incomplete adsorbent aggregation which leads to a decline in Cd ion uptake active surface area may be the reason for the drop in removal efficiency at higher concentration for RFA. 4 g/L adsorbent dose was used for other investigations.

#### 3.2.4. Effect of Mixing Rate

**Figure 12:**
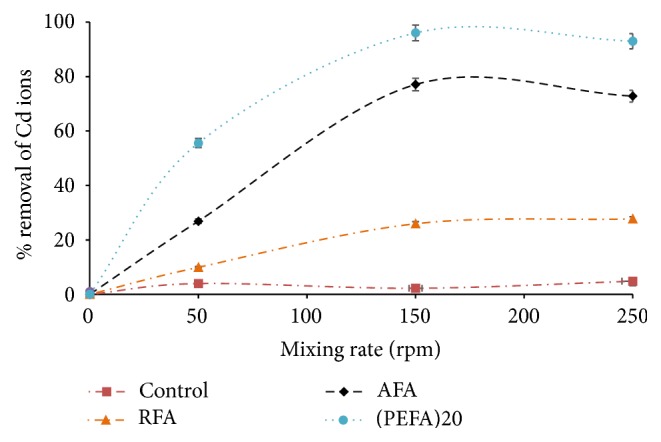
The influence of mixing rate on the removal efficiency of Cd (II) ions on FA based adsorbents (RFA, AFA, and PEFA) as a function of pH value of 9, 2 mg/L metal ion concentration, contact time of 15 min, 4 g/L adsorbent dosage, 50 mL volume of aqueous solution, and 298 K temperature.

The mixing rate ensures that Cd (II) ions are transferred to the active sites by supporting the contact between Cd ions in aqueous solution and adsorbent binding sites [47]. The optimum removal of Cd (II) at pH value of 9 was used to investigate the effect of mixing rate on the adsorption of Cd (II) ion for RFA, AFA, and PEFA.  Figure 12 indicates that the removal of Cd ion increases with mixing rate increase from 50 to 150 rpm. Maximum removal of over 96% was achieved for PEFA, 77% for AFA, and 27% for RFA at 150 rpm with 2 mg/L metal ion concentration, 50 mL volume of aqueous solution, 4 g/L dose of adsorbents, pH value of 9, contact time of 15 min, and 298 K temperature. Afterwards, there was no significant removal achieved above this mixing rate under similar conditions. This observation could be ascribed to improved interaction between the sorption-active sites and Cd ions in aqueous solution with an increase mixing rate [25]. The value of 150 rpm was chosen as optimum mixing rate.

#### 3.2.5. Effect of Initial Concentrations

**Figure 13:**
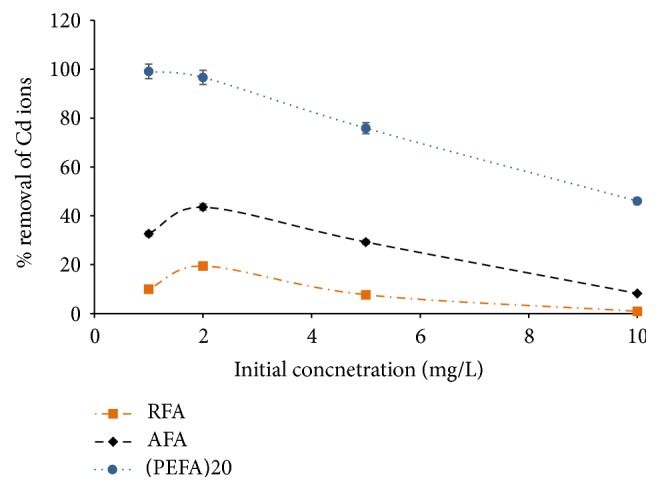
The influence of initial concentration on the removal efficiency of Cd (II) ions on FA based adsorbents (RFA, AFA, and PEFA) as a function of pH value of 9, 150 rpm mixing rate, contact time of 15 min, 4 g/L adsorbent dosage, 50 mL volume of aqueous solution, and 298 K temperature.

Investigating the initial concentration of metal ion is essential in the sorption studies because water and wastewaters contamination does have diverse metal ion concentrations; hence, knowledge of its influence is required for an elaborate sorption investigation [9]. The effect of Cd ion concentration in aqueous solution on its sorption by RFA, AFA, and PEFA was conducted with 4 g/L dose of adsorbent, pH value of 9, 150 rpm mixing rate, 15 min contact time, and 298 K temperature. Initial Cd ions concentrations investigated were varied from 1 to 10 mg/L and their effects on the removal efficiency were established. In Figure 13, it was observed that increasing the initial concentration of Cd (II) ions in solution could cause a decline in the removal efficiency of RFA, AFA, and PEFA. This can be ascribed to bulky quantities of Cd (II) ion with inadequate active sites on the surface of the adsorbents which resulted in increased concentration of Cd (II) ion in the greater part of the aqueous solution and as a result decreasing Cd ion removal efficiency [9, 25]. 

#### 3.2.6. Effect of Temperature

**Figure 14:**
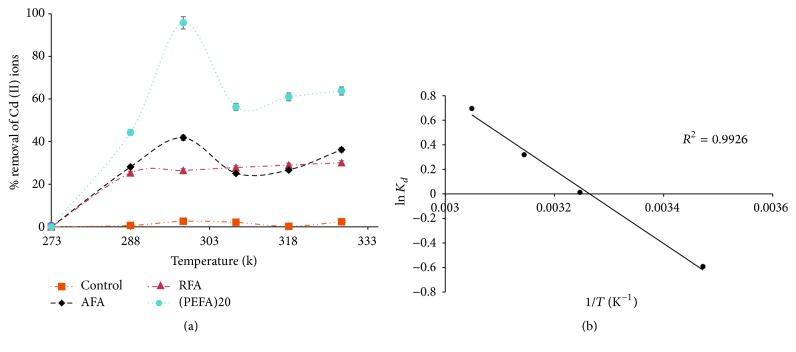
(a) The influence of temperature on the removal efficiency of Cd (II) ions on FA based adsorbents (RFA, AFA, and PEFA) as a function of pH value of 9, 2 mg/L metal ion concentration, 150 rpm mixing rate, contact time of 15 min, 4 g/L adsorbent dosage, and 50 mL volume of aqueous solution. (b) Van Hoff plot for Cd ion sorption at pH value of 9, 2 mg/L metal ion concentration, 150 rpm mixing rate, PEFA, contact time of 15 min, 4 g/L adsorbent dosage, and 50 mL volume of aqueous solution.

Figure 14(a) illustrates Cd ion sorption on RFA, AFA, and PEFA at different temperatures. It can be deduced from the graph that an initial rise in temperature brings about a sharp increase in Cd (II) sorption from 273 to 298 K [38, 48]. This observation could be attributed to the fact that more chemical sites were present as temperature rises from 288 to 298 K to surface component dissociation on PEFA. This also suggests that the adsorption mechanism of Cd (II) ion on PEFA could be chemical sorption in addition to physical sorption as observed for RFA in which sorption increases with an increase in temperature. After a drop in removal efficiency at 308 K, there was a steady increase up till 328 k, suggesting that a high temperature might be a favorable factor in the sorption process as well and indicating that the adsorption is endothermic [9, 38].

To assess the feasibility and spontaneity of sorption process, thermodynamic parameters like Δ*G*° (free energy change), Δ*H*° (enthalpy change), and Δ*S*° (entropy change) were determined as shown in Table 2. Gibbs free energy change of sorption was calculated from the following equation:

(10) $$\begin{array}{c} \Delta G^{{}^{\circ}}=-RT\ln K_{d}, \end{array}$$

where *R* is 8.314 J/mol·K, *T* (K) is the absolute temperature, and *K*_*d*_ is the distribution coefficient expressed as *K*_*d*_ = *q*_*e*_/*C*_*e*_, where *q*_*e*_ is the amount of Cd ion adsorbed at equilibrium and *C*_*e*_ is the concentration of Cd ion in aqueous solution at equilibrium.

(11) ln⁡Kd=−ΔG°RT

(12) ln⁡Kd=−ΔH°RT+ΔS°R.

Equation (12) is known as the Van Hoff equation; the values of Δ*H*° and Δ*S*° were calculated from slope and intercept of the plot of ln⁡*K*_*d*_ against *T*^−1^ (K^−1^) as indicated in Figure 14(b).

### 3.3. Isotherm and Kinetic Studies

#### 3.3.1. Langmuir and Freundlich Isotherm Models

**Figure 15:**
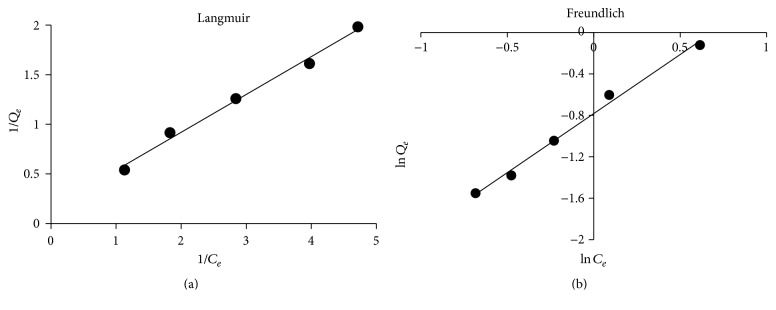
(a) Langmuir adsorption isotherm model. (b) Freundlich adsorption isotherm model.

In order to determine the maximum sorption capacities of PEFA, data gotten at equilibrium for sorption experiment were employed. Figures 15(a) and 15(b) illustrate Langmuir and Freundlich isotherm models for Cd (II) at optimum pH (9). The maximum sorption capacity and adsorption intensity values were calculated from the slope and intercept of the plots between 1/*q*_*e*_ and 1/*C*_*e*_ for Langmuir as in *q*_*m*_  and  *K*_*L*_ [31] and between ln⁡*q*_*e*_ and ln⁡*C*_*e*_ for Freundlich as in *K*_*f*_  and  *n* [49], respectively.  Table 3 shows the correlation coefficient values (*R*^2^) for both Langmuir and Freundlich as well as other parameters. This implies that both models fitted well for the experimental data. Nonetheless, the important features of Langmuir parameters can be applied to further forecast the interaction between the adsorbate and adsorbent with the aid of dimensionless separation parameters (*R*_*L*_) as indicated in the following equation:

(13) $$\begin{array}{c} R_{L}=\frac{1}{1+K_{L}C_{i}}, \end{array}$$

where *K*_*L*_ is Langmuir constant and *C*_*i*_ is Cd (II) ions initial concentration. *R*_*L*_ value gives essential information on sorption nature. *R*_*L*_ value for this study as shown in Table 3 indicates a favorable adsorption process (*R*_*L*_ < 1) for 2 mg/L Cd (II) ion concentration [9, 38, 49]. Adsorption of PEFA can also be explained in terms of surface area coverage in contrast to initial concentration of Cd ion [38]. Langmuir model for surface area coverage of adsorbent surface can be illustrated with aid of the following equation:

(14) $$\begin{array}{c} K_{L}C_{i}=\frac{\theta }{1-\theta }, \end{array}$$

where *θ* is the surface area coverage of adsorbent surface as indicated in Table 3.

#### 3.3.2. Kinetic Studies of Adsorption

As indicated in (7), (8), and (9), kinetic studies of sorption data were evaluated by different kinetic models like pseudo-1st-order model, pseudo-2nd-order model, and intraparticle diffusion [43, 49, 50]. Sorption of cadmium ions was supervised at different period of time. Sorption of Cd (II) ions was calculated from data acquired. To determine the appropriate kinetic model, Cd (II) ion adsorption was plotted against time. These data were fitted into pseudo-1st-order, pseudo-2nd-order, and Weber intraparticle diffusion equations [43].  Table 4 shows that values of *q*_*e*_ and *K*_*i*_ were calculated from *K*_*i*_ (Slope) and ln⁡*q*_*e*_ (intercept) of plot ln⁡(*q*_*e*_ − *q*_*t*_) versus *t*. The correlation coefficient value (*R*^2^ = 0.9802) for pseudo-1st-order model was lower than that of pseudo-2nd-order model. This could be linked to the fact that sorption kinetics take place chemically and involve forces of valency via ions sharing or electron exchange between adsorbent and the adsorbed ions on PEFA [46, 51]. Values of *q*_*e*_ and *K*_2_ were calculated from *q*_*e*_ and 1/*q*_*e*_ (slope) and 1/*K*_2_*q*_*e*^2^_ (intercept) of the plot. The correlation (*R*^2^ = 0.9999) for pseudo-2nd-order model was very strong, pointing towards the fact that sorption of cadmium ions occurred on a monolayer mode, with the assumption that the rate limiting factor could be chemical sorption [38]. This indicated that the cadmium ions were chemically bonded to definite active sites on the surface of PEFA. Weber and Morris' intraparticle diffusion equation was also plotted for *q*_*t*_ against *t*^1/2^ [50]. Values of *K*_*i*_ and *C* were calculated from *K*_*i*_ (slope) and *C* (intercept) as shown in Table 4. Its correlation value (*R*^2^ = 0.8365) was the lowest and the plot intercept did not pass through the origin pointing towards some control of boundary layers and suggesting that intraparticle pore diffusion is not the only rate limiting factor [38]. The intraparticle diffusion equation plot highlights multilinearity, indicating a three-stage process. The initial sharper part is linked to the diffusion of Cd (II) ions via the solution to the external surface of PEFA or boundary layer diffusion of solid molecules [38]. The second part gives description of ion phase, where intraparticle diffusion is a rate limiting factor. The third part is ascribed to the final equilibrium phase. Nonetheless, the intercept of the plot (not shown) fails to pass through the origin, which may be attributed to the difference in the rate of mass transfer in the initial and final phases of sorption [52].

## 4. Conclusion

This study has demonstrated that polyelectrolyte-coated fly ash (PEFA) performed as an excellent adsorbent for Cd (II) ion in aqueous solution. Adsorption of Cd (II) on PEFA surface was dependent on the dosage of adsorbent, pH of the aqueous solution, contact time, Cd (II) initial concentration, and temperature. Optimum conditions for Cd ions removal were found to be at an adsorbent dose of 4 g/L, pH value of 9, 15 min contact time, mixing rate of 150 rpm, 2 mg/L Cd initial concentration, and 298 k temperature. The maximum sorption capacity of PEFA was achieved at 6.40 mg/g with the experimental data fitting well to both Langmuir and Freundlich isotherm models and following pseudo-2nd-order kinetics. The investigation of thermodynamic parameters suggested that the adsorption of Cd (II) ions interaction with PEFA was endothermic and spontaneous and was increasing disorderliness of solute solution interface. This research highlights that fly ash material, a hazardous industrial waste, has a great potential in water treatment application.

## Figures and Tables

**Table 1:** Elemental composition of RFA, AFA, and PEFA as revealed by XRF.

Atomic number	Elements	RFA	AFA	PEFA
14	Silicon (Si)	0	0.76	0.00
15	Phosphorus (P)	1.09	0.66	1.18
16	Sulphur (S)	51.44	86.25	92.50
20	Calcium (Ca)	1.91	2.16	2.23
23	Vanadium (V)	20.22	5.10	1.09
25	Manganese (Mn)	0.1	0.00	0.00
26	Iron (Fe)	11.34	2.10	1.29
28	Nickel (Ni)	13.46	2.95	1.70
30	Zinc (Zn)	0.42	0.00	0.00
42	Molybdenum (Mo)	0.02	0.006	0.005
	Loss on Ignition (LOI)	0.009	0.014	0.005

	*Total*	*100*	*100*	*100*

**Table 2:** Thermodynamic parameters for Cd (II) ions adsorption by polyelectrolyte-coated fly ash (PEFA).

*T* (K)	*K*_*d*_	Δ*G*° (KJ/mol)	Δ*H*° (KJ/mol)	Δ*S*° (J/mol/K)
288	0.55306	1.41818	24.80814	80.98418
298	4.12617	−3.5116		
308	1.01149	−0.0293		
318	1.37544	−0.8428		
328	2.00362	−1.8951		

**Table 3:** Langmuir and Freundlich constants for Cd (II) ion uptake.

	Langmuir constants					Freundlich constants		
	*R* ^*2*^	*K*_*L*_	*Q*_max_	*R*_*L*_	*θ*	*R* ^*2*^	*K*_*f*_	*1*/*n*
PEFA	0.9918	0.4101	6.3939	0.4714	0.5286	0.9924	0.4568	0.878

**Table 4:** Sorption kinetics parameters for Cd (II) ion adsorption by PEFA.

	Pseudo-1st-order			Pseudo-2nd-order			Intraparticle diffusion		
	*q*_*e*_	*K*_1_	*R* ^2^	*q*_*e*_	*K*_2_	*R* ^2^	*K*_*i*_	*C*	*R* ^2^
PEFA	148.9	0.0619	0.9802	0.6052	4.2983	0.999	0.0046	0.8413	0.8365
